# Comparison of the Field Trapping Ability of MgB_2_ and Hybrid Disc-Shaped Layouts

**DOI:** 10.3390/ma17051201

**Published:** 2024-03-05

**Authors:** Michela Fracasso, Roberto Gerbaldo, Gianluca Ghigo, Daniele Torsello, Yiteng Xing, Pierre Bernstein, Jacques Noudem, Laura Gozzelino

**Affiliations:** 1 Department of Applied Science and Technology, Politecnico di Torino, 10129 Torino, Italy; roberto.gerbaldo@polito.it (R.G.); gianluca.ghigo@polito.it (G.G.); daniele.torsello@polito.it (D.T.); laura.gozzelino@polito.it (L.G.); 2 Istituto Nazionale di Fisica Nucleare, Sezione di Torino, 10125 Torino, Italy; 3 UMR 6508, CRISMAT, ENSICAEN, CNRS, UNICAEN, 14050 Caen, France; yiteng.xing@ensicaen.fr (Y.X.); pierre.bernstein@ensicaen.fr (P.B.); jacques.noudem@ensicaen.fr (J.N.)

**Keywords:** MgB_2_ bulk, trapped field, hybrid solutions

## Abstract

Superconductors have revolutionized magnet technology, surpassing the limitations of traditional coils and permanent magnets. This work experimentally investigates the field-trapping ability of a MgB_2_ disc at various temperatures and proposes new hybrid (MgB_2_-soft iron) configurations using a numerical approach based on the vector potential ($\vec{A}$) formulation. The experimental characterization consists in measurements of trapped magnetic flux density carried out using cryogenic Hall probes located at different radial positions over the MgB_2_ sample, after a field cooling (FC) process and the subsequent removal of the applied field. Measurements were performed also as a function of the distance from the disc surface. The numerical modelling of the superconductor required the evaluation of the critical current density dependence on the magnetic flux density (J*_c_*(B)) obtained through an iterative procedure whose output were successfully validated by the comparison between experimental and computed data. The numerical model, upgraded to also describe the in-field behavior of ARMCO soft iron, was then employed to predict the field-trapping ability of hybrid layouts of different shapes. The most promising results were achieved by assuming a hollow superconducting disc filled with a ferromagnetic (FM) cylinder. With such a geometry, optimizing the radius of the FM cylinder while the external dimensions of the superconducting disc are kept unchanged, an improvement of more than 30% is predicted with respect to the full superconducting disc, assuming a working temperature of 20 K.

## 1. Introduction

Superconductors, with their remarkable ability to trap magnetic fields at levels significantly surpassing those achievable with traditional permanent magnets [1,2,3,4], play a pivotal role in advancing magnet technology. This unique property has indeed gathered considerable interest for various engineering applications, such as energy storage systems [5,6,7] high current machines [8,9,10,11], magnetic resonance imaging (MRI), and portable magnets [12], to name just a few. Furthermore, an additional improvement in trapping field ability has been obtained, creating hybrid arrangements wherein ferromagnetic (FM) components are superimposed or integrated into the superconducting (SC) ones [13,14,15,16]. Among superconductors, cost-effectiveness, absence of toxic elements, lack of reliance on rare earth elements, and the ability to transport high critical current across randomly oriented grain boundaries make MgB_2_ a good candidate for trapped field applications [17,18,19]. In addition, recent progresses in bulk fabrication techniques has allowed for the manufacturing of MgB_2_ bulks with high trapped field values even in small-sized samples [20,21]. It was reported that the critical current densities and trapped field depend on the density and homogeneity of the MgB_2_ superconducting material and pinning centers [19,22]. Studies indicated that the homogeneity and density of the superconducting MgB_2_ material and the density of pinning centers can be improved by thermal treatment under high isostatic pressure or by an increase of the density of the MgB_2_ material before the synthesis reaction [23,24,25]. Spark plasma sintering (SPS) is another technique that allows for the preparation of dense and large MgB_2_ samples with a short process time [26,27]. By way of example, trapped field values of 2.12 T were achieved at 5 K (measured above a disc-shaped sample with a radius *R* = 9 mm and a height *h* = 6 mm) [28], 2.4 T at 15.9 K (measured above a disc-shaped sample with *R* = 15 mm and *h* = 6.6 mm) [29], and 3.2 T at 15 K (measured above a disc-shaped sample with *R* = 10 mm and *h* = 1.6 mm) [30]. On the other hand, soft ferromagnetic materials and alloys with high saturation field and magnetic permeability are good candidates for assembling hybrid superconducting/ferromagnetic magnets [31,32].

The development of more and more efficient permanent magnets has also taken advantage of the use of numerical analysis techniques as guidance in their design. The finite element method (FEM) is the most utilized numerical approach to modelling both the superconducting and the ferromagnetic behaviour [33,34,35,36,37,38]. Concerning superconductors, different approaches were developed depending on the specific application. For instance, the time-dependent Ginzburg–Landau (TDGL) equations theory [39] addresses the magnetic flux dynamics at the scale of a vortex and are mostly applied to investigate the mesoscopic regime [40,41,42,43]. Conversely, the behaviour of homogeneous macroscopic samples can be modelled by solving Maxwell’s equations and averaging the electromagnetic fields over a much larger spatial scale [44]. In the latter scenario, a non-linear $\vec{E}-\vec{J}$ characteristic, which accounts for the transition from superconducting to normal state, is typically used [45,46]. Similarly, the ferromagnetic materials are usually described introducing the characteristic B(H) curve or the magnetic permeability, $\mu_{r}$ [47,48,49].

In this work, the field-trapping ability of a MgB_2_ disc grown using the spark plasma sintering (SPS) technique [26,27,50] was experimentally investigated at different working temperatures *T* = 20, 25, and 30 K. After the field cooling (FC) process, the magnetic flux density was measured by means of cryogenic Hall probes located along the sample diameter, and these were taken for different values of the external applied field and as a function of the distance from the disc surface. The possibility of enhancing the trapped field values via a ferromagnetic bulk addition was then studied by employing a numerical approach in order to avoid untargeted and time-consuming experimental trials. To this aim, the vector potential ($\vec{A}$) formulation—presented in [51]—was applied to model the MgB_2_ sample. For this purpose, to properly account for the magnetic field dependence of the critical current density in the MgB_2_ disc, we used an iterative process starting from the equation proposed in [52]. The $J_{c}\left(B\right)$ values obtained at the iteration step (*i* − 1) were entered into the model at the step *i*, until convergence to the measured trapped field curve was obtained. The comparison with the measured data allowed us to validate this numerical procedure. Then, the numerical model was exploited to study some hybrid superconducting–ferromagnetic configurations.

This paper is structured as follows: In Section 2, we present the technique employed for MgB_2_ bulk fabrication (Section 2.1), the experimental setup used to carry out the trapped field measurements (Section 2.2), and the numerical procedure employed to model both the superconducting and hybrid layouts (Section 2.3). The experimental results are provided in Section 3, where we also delved into the procedure for *J_c_*(B) calculation and the comparison between experimental and computational data (Section 3.2). The analysis of the trapped field in the hybrid configurations is then presented in Section 3.3. Finally, the main conclusions and results are summarized in Section 4.

## 2. Materials and Methods

### 2.1. Superconducting Sample

**Figure 1:**
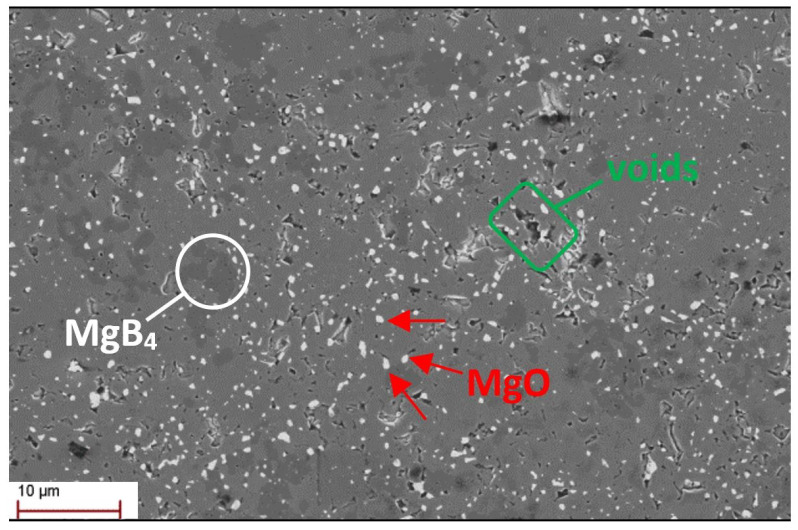
Scanning electron microscopy image of the microstructure of the MgB_2_ disc.

**Figure 2:**
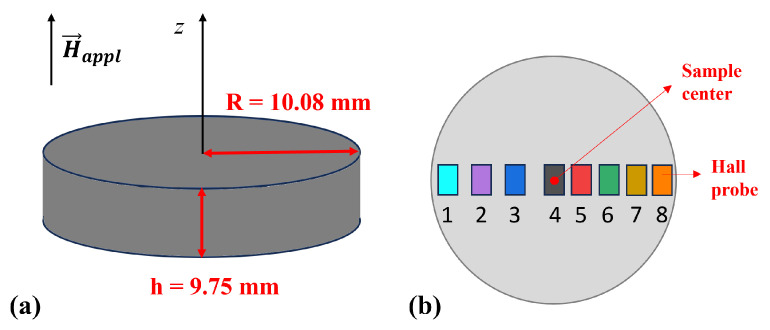
Schematic view of the MgB_2_ disc (**a**) and of the Hall probes’ positions (**b**). The radial positions with respect to the sample center are the following: Hall probe #1: 8.0 mm, #2: 5.75 mm, #3: 3.5 mm, #4: 0.0 mm (sample center), #5: 2.0 mm, #6: 4.0 mm, #7: 6.0 mm, and #8: 8.5 mm.

The MgB_2_ sample was fabricated utilizing the spark plasma sintering system (FCT Systeme GmbH, HD25, Rauenstein, Germany), which is recognized as one of the most efficient technologies for dense MgB_2_ bulk fabrication [27]. The sample was obtained by ex situ procedure using commercial MgB_2_ powder (purity >97%, 100 meshes) purchased from PAVEZYUM Advanced Chemicals, Turkey. The sample was sintered at 1200 °C for 10 min, allowing the production of a highly dense sample [27], while applying an axial pressure of 50 MPa under a dynamic vacuum of $10^{-3}$ bar. The relative density of the sample was 2.6 g/cm^3^, corresponding to 99% of the theoretical value. Figure 1 displays the microstructure of MgB_2_ bulk analyzed using a Carl Zeiss (Supra 55, Oberkochen, Germany) scanning electron microscope (SEM). It confirms the high density of the sample, with four distinct colored zones visible, as mentioned in the previous study [27]. The primary gray zone represents the MgB_2_ phase, while the white regions correspond to MgO phases. The dark gray zones indicate MgB_4_, formed by the evaporation of magnesium during the high sintering temperature process. Additionally, the black regions denote voids present within the sample. The impurities and voids serve as part of the pinning centers landscape in the sample, enhancing the critical current density. The MgB_2_ disc has a height *h* = 9.75 mm and a radius *R* = 10.08 mm (Figure 2a). Its critical temperature is T_*c*_ = 38.5 K.

### 2.2. Experimental Details

The magnetic flux density trapped in the MgB_2_ disc was measured by eight Hall probes, whose disc-shaped active area has a diameter of 300 μm. The Hall probes were distributed along the MgB_2_ disc’s diameter (Figure 2b). The external field ${\vec{H}}_{appl}$ was always applied parallel to the sample axis (henceforth referred to as the *z*-axis) at a temperature, T, of 50 K, then the sample was cooled down to the working temperature and the field was removed. The Hall probes were always positioned to measure the component of the magnetic flux density parallel to the applied magnetic field ($B_{z}$). During cooling and the external field removal, the distance between the Hall probes and the top surface of the sample was kept constant and equal to 1.5 mm. Once the remnant state was reached (i.e., $H_{appl}$ = 0), the magnetic flux density was measured as a function of the distance between the Hall probes and the sample surface. To this aim, the Hall probes were mounted on a holder moving with micrometric resolution along the *z*-axis [53]. All measurements were performed in a vacuum, with the disc placed in tight thermal contact with the second stage of a cryocooler.

### 2.3. Modelling

A numerical model based on the $\vec{A}$-formulation was implemented by the commercial finite-element software COMSOL Multiphysics^®^ 5.6 through the Magnetic Field interface (*mf*). The superconducting sample and the superconducting components in the hybrid layouts investigated by the numerical modelling were always assumed to have a disc shape with the same size as the disc experimentally characterized. The superimposed or embedded ferromagnetic components always preserved the cylindrical symmetry. Therefore, we employed a two-dimensional axisymmetric approach by establishing a cylindrical coordinate system (*r*, ϕ, *z*) with the origin placed in the center of the sample’s top surface. At a considerable distance from the MgB_2_ disc, the magnetic flux density, *B*, was expected to be parallel to the disc axis and have a magnitude equal to $\mu_{0}H_{appl}$ decreasing with a ramp rate of 0.35 T/s.

The electromagnetic characteristics of the superconducting, the ferromagnetic, and the surrounding domains are described through Maxwell’s equations, which are formulated using the magnetic vector potential $\vec{A}$:

(1) $$\vec{E}=-\frac{\partial \vec{A}}{\partial t}$$

(2) $$\vec{B}=\nabla \times \vec{A}$$

Due to the chosen axisymmetric approach, the vector potential has a single component, $A_{\phi }$, allowing us to rewrite Equations (1) and (2) as:

(3) $$E_{\phi }=-\frac{\partial A_{\phi }}{\partial t}$$

(4) $$\vec{B}=-\frac{\partial A_{\phi }}{\partial z}{\hat{u}}_{r}+\frac{\partial A_{\phi }}{\partial r}{\hat{u}}_{z}$$

with ${\hat{u}}_{r}$ and ${\hat{u}}_{z}$ being the unit vectors along the *r*- and *z*-directions, respectively.

The superconducting domain is distinguished from the others by the presence of a local current density, *J*, which is dependent on the local electric field, *E*, through a hyperbolic tangent function, as suggested by M. Solovyov et al. [51]. This dependence can be considered a smooth approximation of the step-wise *E-J* relation predicted in the critical state model [45]. Consequently, the 2D *E-J* relation takes the form of:

(5) $$J_{\phi }=J_{c}\left(B\right)\tanh\left(\frac{E_{\phi }}{E_{0}}\right)$$

with $E_{\phi }=-\frac{\partial A_{\phi }}{\partial t}$, $E_{0}$ being the threshold electric field used to define the critical current density (here assumed to be equal to $10^{-4}$ V/m) and *J_c_*(*B*) being the local critical current density, calculated with the procedure described in Section 3.2.

Thanks to a relative permeability exceeding 7500 at low fields, a saturation magnetic flux density ≈ 2.3 T, and its negligible hysteresis losses, we chose ARMCO pure iron as ferromagnetic material and employed its B–H characteristic curve to model the material properties [54].

## 3. Results and Discussion

### 3.1. Experimental Results

**Figure 3:**
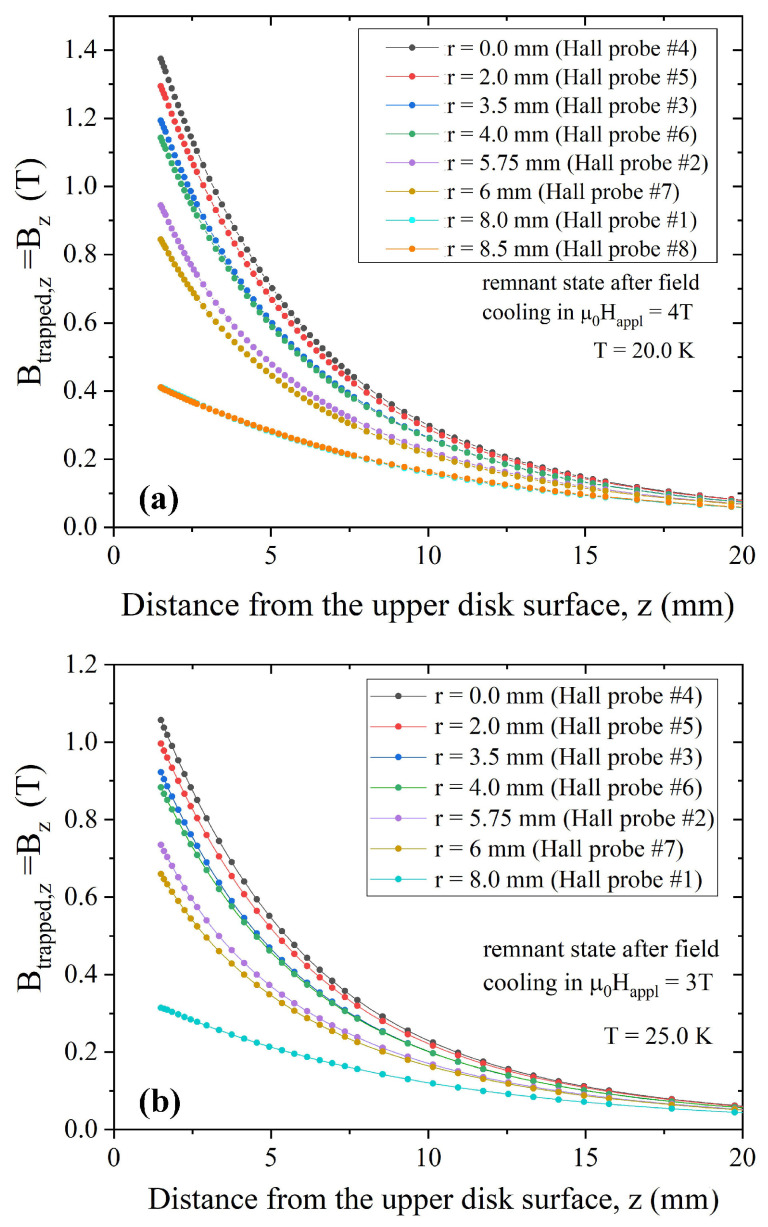

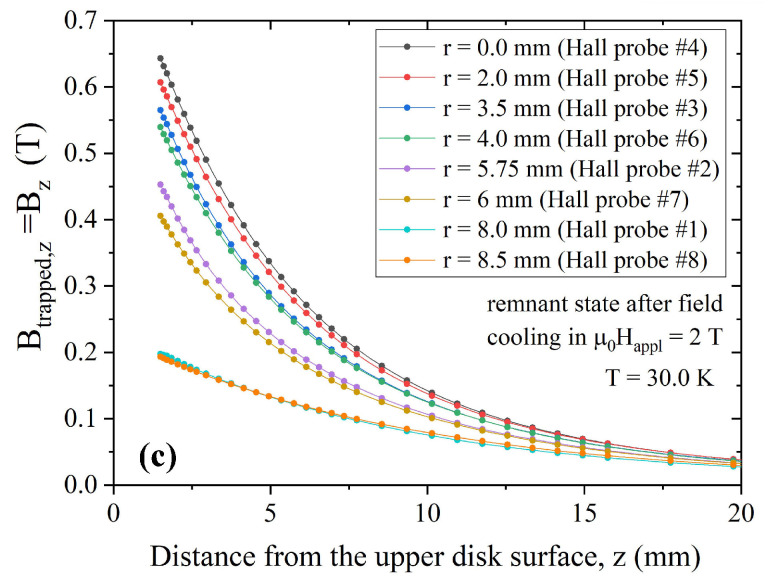
Trapped field values measured by the Hall probes at the radial positions reported in the figure legends as a function of the distance from the top surface of the MgB_2_ disc. The data were obtained at T = 20 K (**a**), T = 25 K (**b**), and T = 30 K (**c**).

We investigated the field-trapping ability of the disc in the temperature range from 20 to 30 K, with ${\vec{B}}_{trapped}$ = $\vec{B}-\mu_{0}{\vec{H}}_{appl}$. Figure 3 shows the z-component of the trapped field measured in the remnant state (i.e., after the complete removal of the applied field—note that in this case $B_{trapped,z}$ = $B_{z}$ − $\mu_{0}H_{appl}$ = $B_{z}$) as a function of the distance *z* from the sample top surface and for different radial positions. The data were collected after field cooling in an external field $\mu_{0}H_{appl}$ = 4 T, $\mu_{0}H_{appl}$ = 3 T, and $\mu_{0}H_{appl}$ = 2 T at 20 K (Figure 3a), 25 K (Figure 3b), and 30 K (Figure 3c), respectively. Focusing on the values measured at the center of the disc (*r* = 0) and at a distance from the disc surface *z* = 1.5 mm, at 20 K, we obtained trapped field values of $B_{trapped,z}$ = 1.37 T, while $B_{trapped,z}$ = 1.05 T and $B_{trapped,z}$ = 0.64 T were obtained at temperatures T = 25 K and T = 30 K, respectively. The achieved results are comparable with those obtained in literature, considering samples with an aspect ratio (*r/h*) and experimental conditions similar to ours and reported in [19,55]. Notably, the $B_{z}$ curves measured at positions #1 (*r* = 8 mm, left side with respect to the disc’s center, see Figure 2b) and #8 (*r* = 8.5 mm, right side) superimpose each other. This could be due to a local inhomogeneity in the sample composition or to a slight lateral shift of the Hall probes due to a small uncertainty of the Hall probes position inside their packages. Indeed, the $B_{z}$ curves measured at positions #2 (*r* = 5.75 mm, left side with respect to the disc’s center) and #7 (*r* = 6 mm, right side) are well separated, thus supporting the first supposition.

### 3.2. Critical Current Density Evaluation and Validation of the Numerical Model

**Figure 4:**
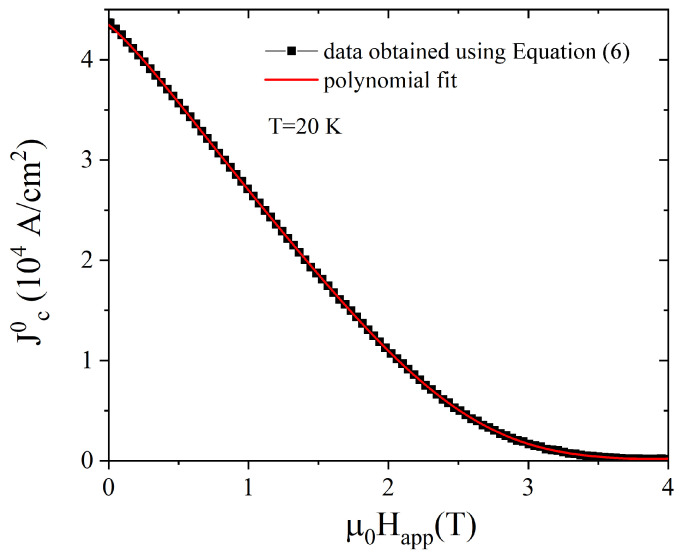
$J_{c}$ dependence on the applied magnetic field at T = 20 K obtained from the $B_{trapped,z}$ vs. $\mu_{0}H_{appl}$ curve measured at *r* = 0 (Hall probe #4) applying Equation (6) (black symbols) and calculated with a polynomial fit (see text—red solid line).

To properly describe the superconductor behaviour, a suitable *J_c_*(*B*) equation is needed. Taking advantage of the sample shape, the first step was an approximate estimation of the critical current density from the trapped magnetic flux density, $B_{trapped,z}$, measured along the disc axis (position #4) as a function of the applied field, using the equation provided by Chen et al. [52]:

(6) $$J_{c}^{0}=\frac{2B_{trapped,z}}{\mu_{0}}\frac{1}{\left(z+h\right)\ln\left(\frac{R}{z+h}+\sqrt{1+\frac{R^{2}}{{\left(z+h\right)}^{2}}}\right)-z\ln\left(\frac{R}{z}+\sqrt{1+\frac{R^{2}}{z^{2}}}\right)}$$

where *R* and *h* are the radius and the height of the disc, respectively, and *z* is the distance of the Hall probe #4 from the top of the sample. It is worth mentioning that since Equation (6) refers to trapped flux density values measured on the sample axis, the flux density magnitude here coincides with its *z* component. First, we applied Equation (6) to the measured $B_{trapped,z}$ at 20 K, obtaining the $J_{c}^{0}$ values plotted in Figure 4.

**Figure 5:**
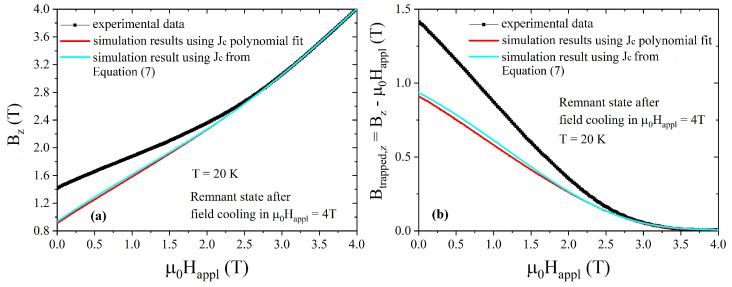
Comparison between the experimental (symbols) and computed (lines) magnetic flux density (**a**) and trapped field (**b**) values, both plotted as a function of the applied magnetic field. The computed curves were obtained using the $J_{c}\left(B\right)$ polynomial law plotted in Figure 4 (red curve) and the exponential $J_{c}$(B) relationship reported in Equation (7) (cyan curve). The measurement was carried out by Hall probe #4 located on the disc’s axis at 20 K and 1.5 mm above the disc surface, decreasing the applied field after field cooling in an external field $\mu_{0}H_{appl}$ = 4 T.

The $J_{c}$ curve was then fitted using a polynomial law, and the resulting $J_{c}$ dependence on magnetic field was entered into Equation (5) and used to recalculate the magnetic flux density. As can be seen in Figure 5, the agreement between the experimental and as-calculated magnetic flux density curves (red solid line) only occurs at high magnetic fields, i.e., above the full penetration field, that for this sample, at 20 K is ≈ 2.5 T. This could be related to the fact that Chen’s method, based on the Bean model hypothesis, assumes a constant critical current density. Indeed, as shown by Chen and Goldfarb [56], the Bean model could give deceptive results when used to estimate the critical current density below the full penetration field, i.e., when $J_{c}$ is strongly dependent on *H_appl_*.

To find a $J_{c}$(*B*) dependence that could reasonably reproduce the experimental trapped field values also at low applied fields, following the procedure employed in [57] we fitted the high-field range (2.5–4 T) of the curve reported in Figure 4—i.e., the field range above the full penetration field, where a good agreement between experiment (black symbols) and simulation (red solid lines) was clearly visible in Figure 5—by the exponential relation reported below:

(7) $$J_{c}\left(\mu_{0}H_{appl}\right)=J_{k}\exp\left[-{\left(\frac{\mu_{0}H_{appl}}{B_{0}}\right)}^{\gamma }\right]$$

where $J_{k}$, *B*_0_ and γ are fitting parameters. However, at low field, the agreement between the experimental and the computed data was still poor, as one can see in Figure 5. This could be due to the small portion of the experimental curve considered for the fit.

To overcome this problem, we modified the procedure by introducing intermediate steps based on the following iterative procedure:

- Starting from the curve $B_{trapped,z}$ vs. $\mu_{0}H_{appl}$ reported in Figure 5, we evaluated the difference between the experimental and computed trapped fields, i.e., $\Delta B_{trapped,z}^{i}$ (= $B_{trapped,z,exp}$ − $B_{trapped,z,comp}^{i}$), where $B_{trapped,z,exp}$ is the measured magnetic flux density presented in Section 3.1 and $B_{trapped,z,comp}$ is the computed values obtained fitting the $J_{c}\left(B\right)$ curve with a polynomial fit. In the first iteration, the index *i* was set to 0;
- We applied Chen’s formula (i.e., Equation (6)) to evaluate

(8) $$\Delta J_{c}^{i}=\frac{2\Delta B_{trapped,z}^{i}}{\mu_{0}}\frac{1}{\left(z+h\right)\ln\left(\frac{R}{z+h}+\sqrt{1+\frac{R^{2}}{{\left(z+h\right)}^{2}}}\right)-z\ln\left(\frac{R}{z}+\sqrt{1+\frac{R^{2}}{z^{2}}}\right)}$$

- We built a new $J_{c}^{i+1}$ vs. $\mu_{0}$$H_{appl}$ curve adding $\Delta J_{c}^{i}$ to the $J_{c}^{i}$ values calculated in the previous step;
- We fitted the $J_{c}^{i+1}$ vs. $\mu_{0}$$H_{appl}$ by a polynomial law;
- We inserted the new $J_{c}^{i+1}$ vs. $\mu_{0}$$H_{appl}$ dependence in the numerical procedure and achieved new $B_{z,comp}^{i+1}$ and $B_{trapped,z,comp}^{i+1}$ vs. $\mu_{0}$$H_{appl}$ curves. We repeated the whole procedure several times (i=0,1,…,8).

**Figure 6:**
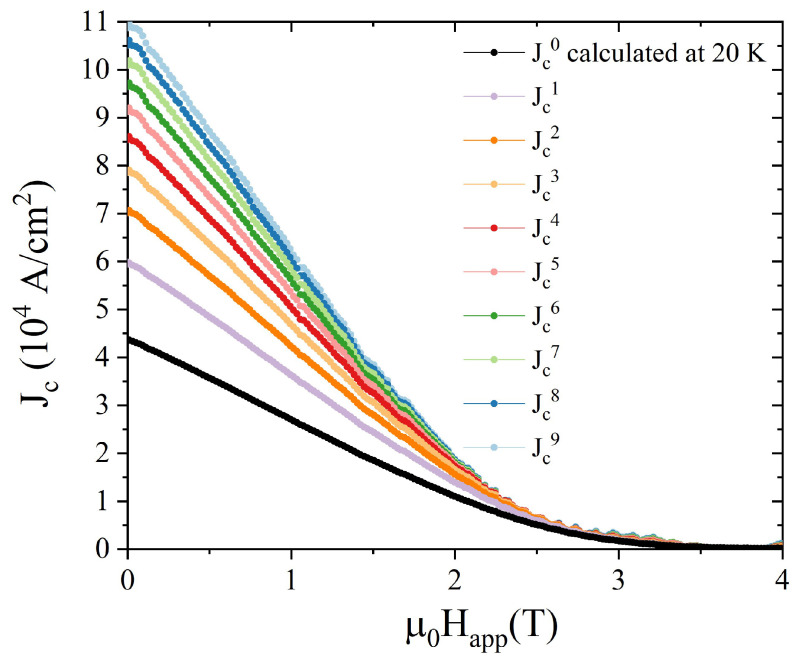
Comparison among $J_{c}^{0}$ and $J_{c}^{i+1}$ vs. $\mu_{0}H_{appl}$ curves obtained from the values of the trapped field assessed by measurement applying Equation (6) (black curve) and through computations (coloured curves). The latter represent the results obtained after each iteration of the iterative process presented in the main text. The final aim was to define a $J_{c}$(B) suitable to reproduce the experimental data, which was identified in the $J_{c}^{9}$ curve (light-blue curve).

Figure 6 shows the $J_{c}^{0}$ and $J_{c}^{i+1}$ vs. $\mu_{0}$$H_{appl}$ curves obtained after each iteration.

**Figure 7:**
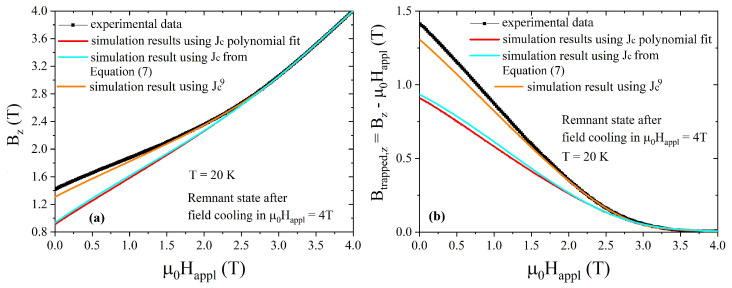
Comparison between the experimental (symbols) and numerically computed (lines) magnetic flux density (**a**) and trapped field (**b**) values, both plotted as a function of the applied magnetic field. The computed curves were obtained using the $J_{c}\left(B\right)$ polynomial law plotted in Figure 4 (red curve), the exponential $J_{c}\left(B\right)$ in Equation (7) (cyan curve), and the $J_{c}^{9}\left(B\right)$ polynomial law extracted following the procedure described in the main text (orange curve). The measurement was carried out at 20 K, decreasing the applied field after field cooling in an external field $\mu_{0}H_{appl}$ = 4T. Data refer to Hall probe #4 position, placed on the sample’s axis, 1.5 mm above the disc top surface.

At this point, the simulated $B_{z,comp}^{9}$ and $B_{trapped,z,comp}^{9}$ curves reproduces the experimental data in a larger range of applied fields (Figure 7), even though a small difference still occurs at low field.

**Figure 8:**
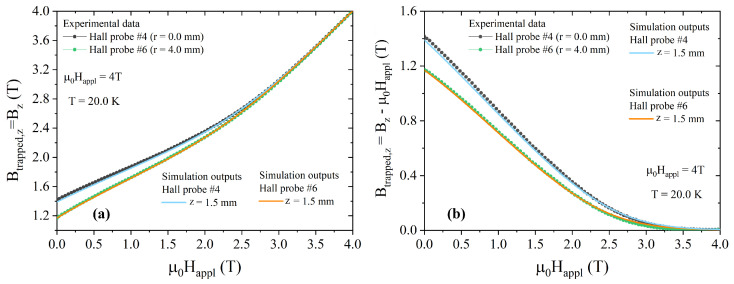
Comparison between the magnetic flux density (**a**) and trapped field (**b**) measured by Hall probes #4 and #6 at 20 K when decreasing the external field after field cooling in $\mu_{0}H_{appl}$ = 4 T (symbols) and the corresponding computed values (lines).

**Figure 9:**
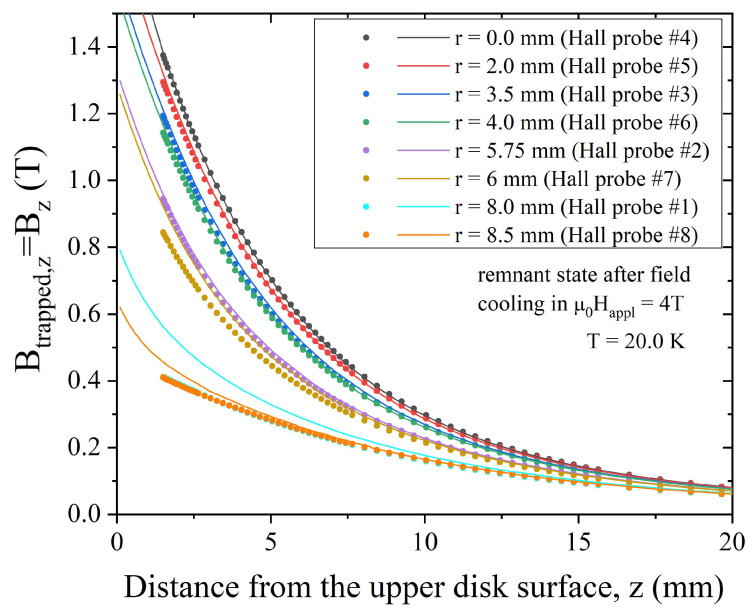
Comparison between the trapped magnetic flux density measured by all the Hall probes as a function of the distance from the top surface of the disc, at 20 K, in remnant state after field cooling in $\mu_{0}H_{appl}$ = 4 T (symbols), and the corresponding values computed by numerical simulations (lines).

To overcome this discrepancy, we fitted the $J_{c}^{9}$ vs. $\mu_{0}H_{appl}$ curve by Equation (7), obtaining the following fitting parameters: $J_{k}$ = 1.86 × 10^5^ A/cm^2^, B_0_ = 1.21 T, and γ = 1.66 (quite comparable to the values obtained by Fujishiro et al. [58] in MgB_2_ bulk disc at T = 20 K). Finally, Equation (7), with the above-mentioned fitting parameters $J_{k}$, $B_{0}$, and γ, was entered into Equation (5) and employed to calculate the final magnetic flux density through the simulations. This procedure provided a very good agreement between the experimental and computed data for the innermost radial positions, as shown in Figure 8. The same excellent agreement was found by comparing the trapped field data measured and computed in remnant state as a function of the distance from the sample top surface (Figure 9). On the contrary, some differences occur for radial positions *r* ≥ 6 mm. This could support both the hypothesis of small inhomogeneities in the $J_{c}$ bulk distributions or small uncertainty of the Hall probes’ positions inside their packages.

**Figure 10:**
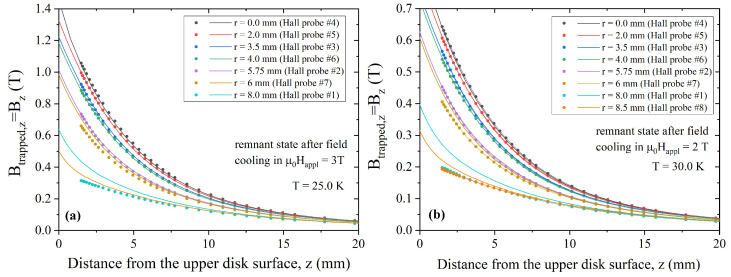
Comparison between the magnetic flux density measured by all of the Hall probes as a function of the distance from the top surface of the disc, at T = 25 K (**a**), and T = 30 K (**b**) after field cooling in an applied field of $\mu_{0}H_{appl}$ = 3 T and 2 T, respectively (symbols), and the corresponding values computed by numerical simulations (lines).

Lastly, the whole procedure was repeated starting from trapped field data obtained at 25 and 30 K, after field cooling in an external field $\mu_{0}H_{appl}$ = 3 T and $\mu_{0}H_{appl}$ = 2 T, respectively. As can be seen in Figure 10, for both the temperatures, a good agreement between the experimental and computed data was achieved. Table 1 summarized the fitting parameters entered into Equation (7) to account for the $J_{c}$(B) dependence.

This agreement between the experimental and calculated results allowed us to verify the feasibility of this numerical model to predict trapping field values and to exploit it to investigate the performance of new hybrid superconducting–ferromagnetic layouts of permanent magnets.

### 3.3. Trapped Fields in Hybrid Samples

**Figure 11:**
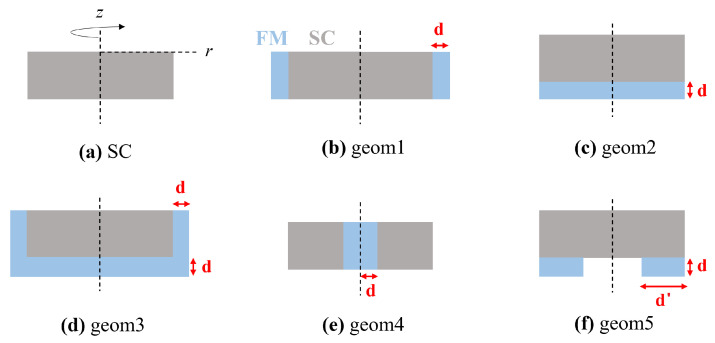
Schematic view of the hybrid layouts numerically investigated. The superconducting components (grey) are discs with the same size as the disc characterized experimentally, except the layout *geom4*, as detailed in the main text. The dimensions of the ferromagnetic components (cyan) are characterized by two recurring values d=2.43 mm and $d^{\prime }=5.04$ mm.

Starting from the layouts and results reported in [31] by Philippe et al., and in [32] by Li et al., we applied the as-validated model to the study of new hybrid configurations of MgB_2_-soft iron. In Figure 11, the cross-section of the investigated layouts is depicted. For the sake of consistency, the MgB_2_ disc has the same dimensions as those experimentally characterized except layout, *geom4* which consists of a hollow superconducting disc filled with a ferromagnetic cylinder. The outer R*_out_* and inner R*_in_* radius of the hollow disc satisfy the condition R*_out_* − R*_in_* = R, being R the radius of the disc characterized experimentally. Conversely, the ferromagnetic bulk is characterized by two recurring dimensions, d=2.43 mm and $d^{\prime }=5.04$ mm.

We focused on the trapped field capabilities of these hybrid configurations, assuming a working temperature of 20 K and a field cooling process in an applied magnetic field $\mu_{0}H_{appl}$ = 4 T. Data here compared were calculated in remnant state, i.e., $B_{z}$ = $B_{trapped,z}$. Note that from this point forward, for the sake of brevity, $B_{z}$ and $B_{trapped,z}$ always refer to the computational values. The dependence of the trapped flux density on the radial position was assessed at 1 mm above the top surface and 1 mm below the bottom surface of all the hybrid arrangements. This choice was made in order to enable a coherent comparison with data reported in the literature [31,32], and takes into consideration that the qualitative behaviour of the trapped field profiles remains unaffected and that the samples’ performances vary only ≈10% between 1 mm and 1.5 mm, which is the minimum distance reached in the experiment (see previous sections).

**Figure 12:**
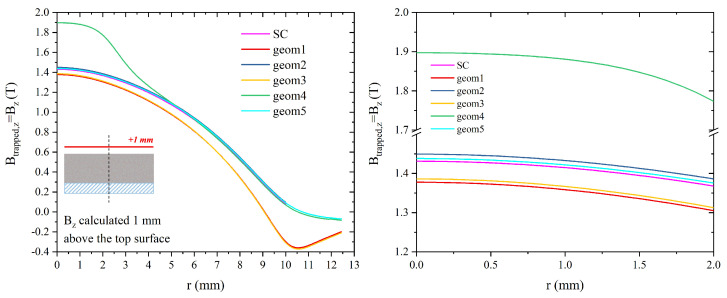
(**Left**) Magnetic flux density $B_{z}$ calculated 1 mm above the top surfaces of the hybrid configurations. (**Right**) magnification of the $B_{z}$ values in the zone close to the disc’s axis.

Figure 12 shows a comparison of the magnetic flux density calculated 1 mm above the top surface for all the hybrid arrangements as a function of the distance from the layout’s symmetry axis (i.e., *z*-axis). For the sake of clarity, a magnification of the zone close to the axis is plotted in the right panel of the figure. Notably, the hybrid layouts referred to as *geom1* and *geom3* exhibit a trapped field degradation in comparison to the superconducting disc alone, while layouts *geom2* and *geom5* provide a slightly better field-trapping ability than the MgB_2_ disc. Conversely, a B*_z_* peak value of 1.90 T is achieved with configuration *geom4* in correspondence to the *z*-axis. It is worth mentioning that the positive effect of the FM addition in this layout persists all along the disc radius up to about r=R/2. The results are partially comparable to those obtained in the literature for similar hybrid configurations. Specifically, in references [32,48], slight improvements were reported for all the proposed hybrid configurations, including the configurations we defined as *geom1* and *geom3*. This different behaviour could likely be attributed to the superior initial performance of the MgB_2_ magnet compared to that obtained with YBCO magnets, which could make the role of the FM layer not so effective for these configurations.

**Figure 13:**
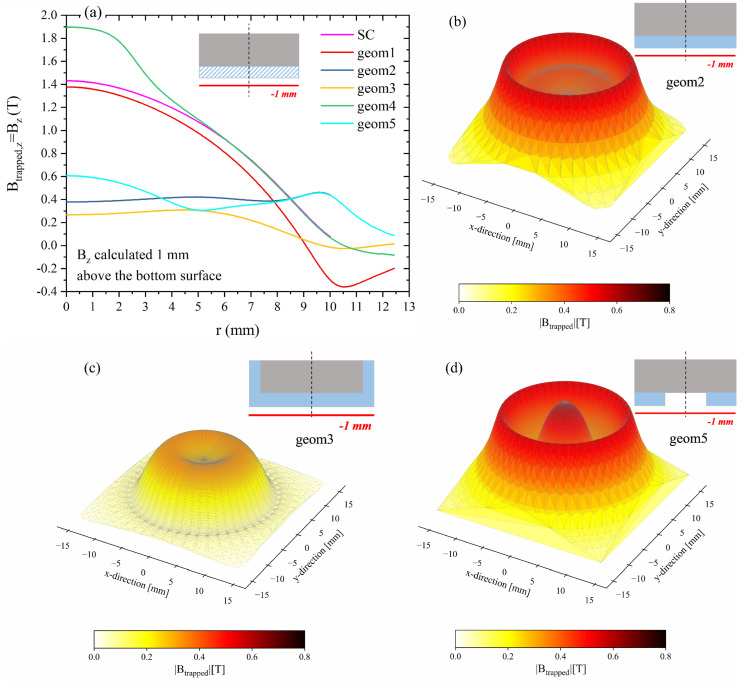
(**a**) $B_{trapped,z}$ dependence on the radial position calculated 1 mm above the surface with the FM layers. (**b**–**d**) 3D maps of the trapped field magnitude calculated 1 mm from the surface with the FM layers for *geom2*, *geom3*, and *geom5*, respectively.

However, in agreement with [31,32], the addition of an FM layer also produces a strong decrease in the trapped magnetic flux density near the surface covered with the FM layers of layouts *geom2*, *geom3*, and *geom5*. In these samples, deterioration of the field-trapping ability of up to 74, 81, and 58%, respectively, was observed, as shown in Figure 13, where the $B_{trapped,z}$ values are plotted as a function of the radial position (a) and the 3D maps of the magnitude of trapped field are depicted for *geom2* (b), *geom3* (c), and *geom5* (d). As expected, this asymmetric behaviour does not occur in layouts *geom1* and *geom4* that show the same field-trapping ability both above and below the sample.

**Figure 14:**
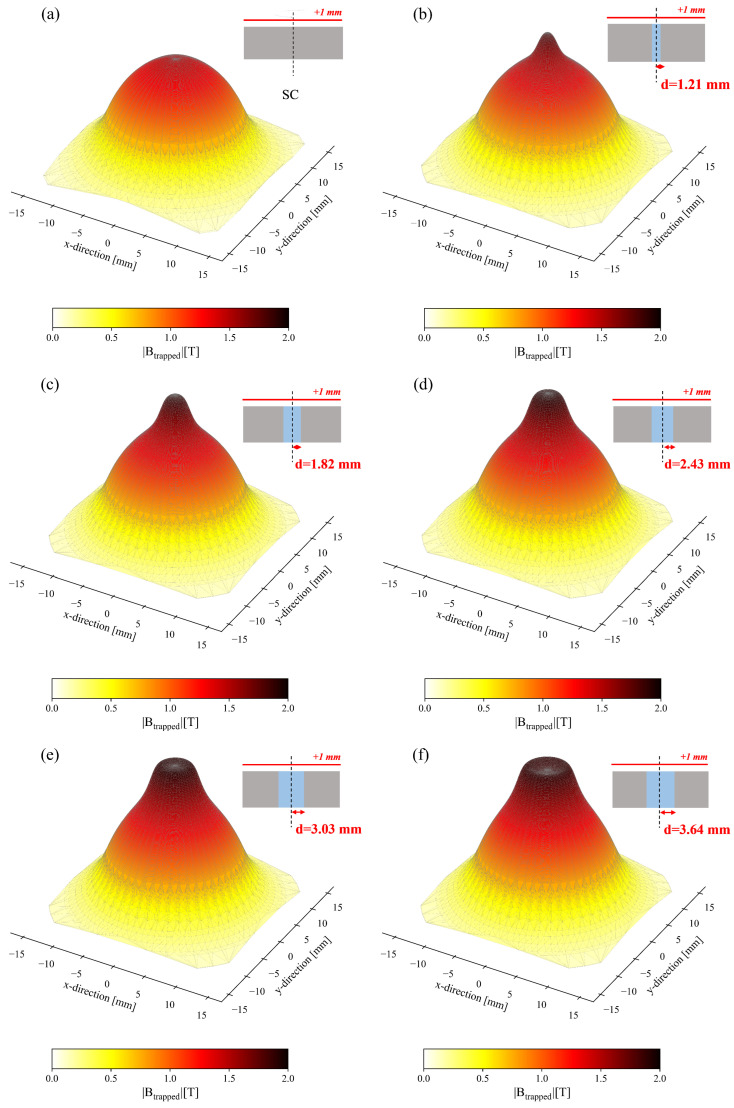
These are 3D maps of the trapped flux density magnitude calculated 1 mm above the top surfaces of superconducting disc (**a**) and layout *geom4*, the latter as the radius of the ferromagnetic cylinder increases: d=1.21 mm (**b**), d=1.82 mm (**c**), d=2.43 mm (**d**), d=3.03 mm (**e**), and d=3.64 mm (**f**).

Based on these results, it is evident that arrangement *geom4* exhibits the highest efficiency among those investigated. Consequently, we proceeded to examine how the trapped field profile varies as the diameter of the hole in the superconductor increases. In all cases, we assumed that the hole was completely filled with iron and kept constant the outer radius of the superconducting disc. Specifically, four other values were considered for the radius of the ferromagnetic cylinder in addition to d=2.43 mm: d=1.21 mm, d=1.82 mm, d=3.03 mm, and d=3.64 mm. Figure 14 shows the comparison of the magnitude of the trapped field values calculated for the superconductor alone (Figure 14a) and the layout *geom4*, the latter as the radius of the FM component increases (Figure 14b–f).

**Figure 15:**
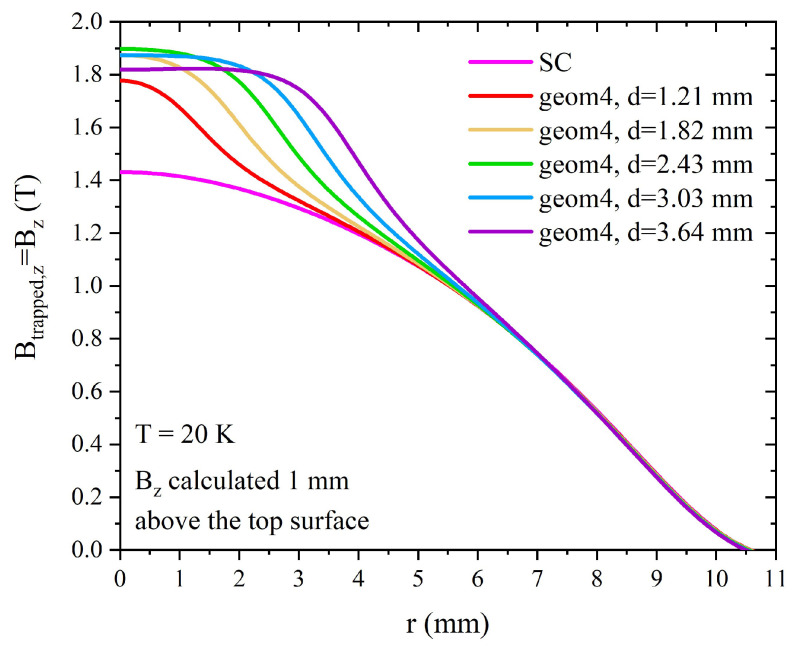
Magnetic flux density $B_{z}$ calculated 1 mm above the top surfaces of layouts named *geom4* for different values of the FM cylinder radius. The outer radius of the hollow superconducting disc surrounding the FM was kept constant and equal to 10.08 mm.

The effect of the ferromagnetic component addition in this layout is twofold. First, the trapped field value at the center of the disc (*r* = 0) improves with the addition of the ferromagnetic cylinder, reaching a maximum value of $B_{trapped}$ = 1.89 T with a ferromagnetic radius of 2.43 mm (Figure 15). Notably, a larger radius of the FM cylinder (d = 3.03 mm and d = 3.64 mm), leads to a decrease of the trapped flux density, the value of which nevertheless remains greater than that obtained with the superconducting disc alone. Concurrently, the addition of the FM layer extends the overall capability of the sample to trap higher values of magnetic flux, even at a radial distance from the disc axis greater than the FM cylinder radius. For instance, considering the maximum $B_{z}$ value of the SC disc at *r* = 0, $B_{z,max}^{SC}$ = 1.43 mm, the same value is reached at *r* = 2.19 mm, *r* = 2.70 mm, *r* = 3.20 mm, *r* = 3.65 mm, and *r* = 4.10 mm when employing FM cylinder with a radius of d=1.21 mm, d=1.82 mm, d=2.43 mm, d=3.03 mm, and d=3.64 mm, respectively.

**Figure 16:**
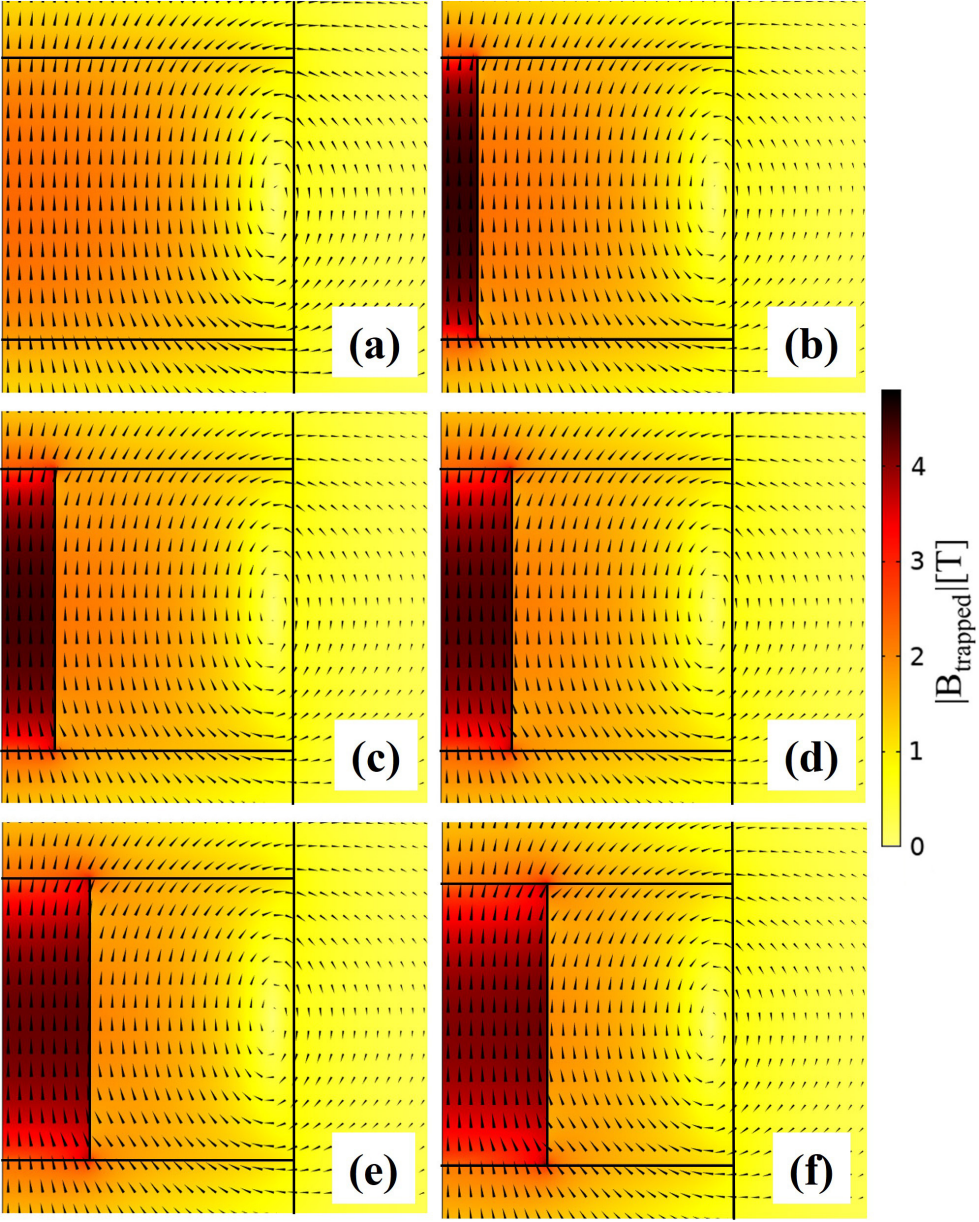
Magnitude of the trapped flux density values (colour maps) and distribution of the magnetic flux lines for the SC disc (**a**) and the five layouts *geom4* with an FM cylinder of radius d=1.21 mm (**b**), d=1.82 mm (**c**), d=2.43 mm (**d**), d=3.03 mm (**e**), and d=3.64 mm (**f**) calculated at T = 20 K.

The improvement of the trapped field efficiency in all layouts *geom4* can be ascribed to a change of the magnetic flux line distribution induced by the FM addition. Figure 16 shows the magnitude of the trapped flux density values (colour maps) and the distribution of the magnetic flux lines for the SC disc (a) and the five layouts *geom4*. As can be seen, the ferromagnetic component attracts the magnetic flux lines [59], thus inducing their accumulation in the region closest to the *z*-axis, which in turn results in an increased trapped flux. Nevertheless, it is worth mentioning that the effect of the FM inclusion is not simply additive to the value of magnetic flux trapped in the SC disc. Indeed, using only an FM cylinder with a radius *d* = 2.43 mm, a trapped field $B_{trapped}$ = 0.006 T is obtained in remnant state at *r* = 0.

**Figure 17:**
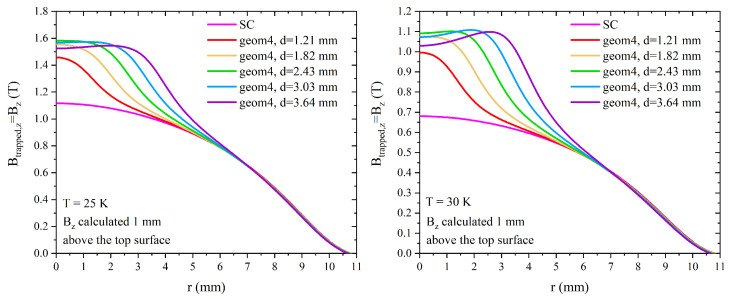
Magnetic flux density $B_{z}$ calculated 1 mm above the top surfaces of the layouts *geom4* for different values of the FM cylinder radius and assuming a working temperature of T = 25 K (**left**) and T = 30 K (**right**).

The improvement and radial behaviour of the trapped flux density achieved with this layout, assuming a working temperature of 20 K, were also confirmed using the $J_{c}$($\mu_{0}H_{appl}$) dependence reported in Equation (7) with the fitting parameters found at 25 K and 30 K (see Table 1). Figure 17 illustrates the $B_{z}$ values calculated 1 mm above the top surfaces of the hybrid geometries at operating temperatures of 25 K (left) and 30 K (right). In both the cases, one can again observe a maximum value of trapped flux density when the radius of the ferromagnetic cylinder is 2.43 mm. Nevertheless, the inclusion of the FM component strongly enhances the trapped flux density as at 20 K, expanding its positive effects also on a radial distance larger than the FM cylinder radius.

**Figure 18:**
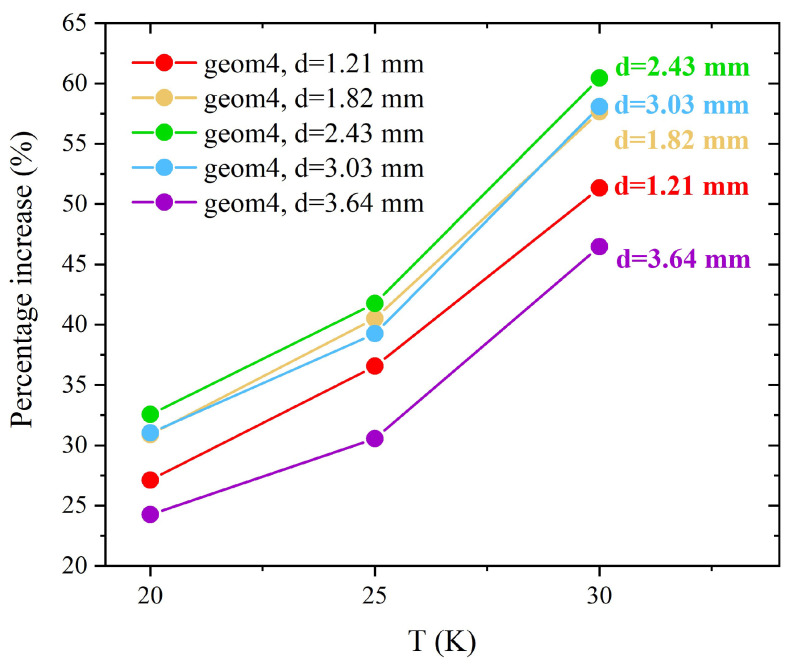
Percentage increase of the magnetic flux density trapped in remnant state by the five layouts *geom4* with respect to the magnetic flux density trapped by the superconducting disc alone.

As expected, the influence of the FM cylinder becomes more pronounced as the temperature rises, mitigating the decline in superconductor performance. This trend is evident in Figure 18, where the percentage increase of the magnetic flux density trapped in the remnant state by the various layouts *geom4* with respect to the flux density trapped by the only superconducting disc is plotted as a function of the operating temperatures. At all of the investigated temperatures, d=2.43 mm is the optimal FM radius above which the performance of the hybrid configurations begins to deteriorate. By way of example, at T = 30 K, a maximum increment of 60.4% is obtained using *geom4* with d=2.43 mm (green curve), while increments of 58 and 46.4% are obtained employing *geom4* with d=3.03 mm (cyan curve) and d=3.64 mm (purple curve), respectively.

## 4. Conclusions

In this study, we investigated the field-trapping ability of a MgB_2_ disc fabricated using the spark plasma sintering (SPS) technique. After the field cooling process, the trapped flux density was measured at various temperatures (20 K, 25 K, and 30 K) during external field removal and in remnant state as a function of the distance from the disc surface. The analysis evidenced the sample’s ability to trap magnetic flux density up to 1.37 T in the remnant state at 20 K and 1.5 mm above the disc surface.

New hybrid (MgB_2_—soft iron) configurations of permanent magnets were then investigated employing a modelling approach based on the $\vec{A}$-formulation. The model feasibility of reproducing the magnetic flux density distribution was verified by comparing experimental and computational data achieved on the superconducting disc, with a good agreement between the two datasets. Various hybrid layouts were assessed, and the layout consisting of a hollow superconducting disc filled with an FM cylinder turned out to be the most efficient geometry. Focusing on this configuration, a maximum $B_{trapped}$ of 1.89 T was achieved with an FM cylinder having a radius of 2.43 mm.

The analysis of the percentage increase of the trapped flux density values also highlighted the temperature-dependent effect of the FM component addition and the importance of its size in order to maximize the permanent magnet performance.

## Figures and Tables

**Table 1:** Fitting parameters of Equation (7) obtained for a working temperature of 20, 25, and 30 K.

T [K]	$J_{k}$ [A/cm^2^]	$B_{0}$ [T]	γ
20	1.86 × 10^5^	1.21	1.66
25	1.48 × 10^5^	0.93	1.80
30	8.74 × 10^4^	0.59	2.00

## Data Availability

The data presented in this article will be shared on reasonable request from the corresponding author.
